# Potential of Cooperage Byproducts Rich in Ellagitannins to Improve the Antioxidant Activity and Color Expression of Red Wine Anthocyanins

**DOI:** 10.3390/foods8080336

**Published:** 2019-08-09

**Authors:** María Luisa Escudero-Gilete, Dolores Hernanz, Celia Galán-Lorente, Francisco J. Heredia, María José Jara-Palacios

**Affiliations:** 1 Food Colour and Quality Laboratory, Área de Nutrición y Bromatología, Facultad de Farmacia, Universidad de Sevilla, 41012 Sevilla, Spain; 2 Department of Analytical Chemistry, Facultad de Farmacia, Universidad de Sevilla, 41012 Sevilla, Spain

**Keywords:** oak shavings, phenolic compounds, model solutions, antioxidant activity, copigmentation

## Abstract

Cooperage byproducts are an important source of phenolic compounds that could be used for wine technology applications. The effects of the addition of two types of oak wood shavings (American, AOW, and Ukrainian, UOW) on the antioxidant activity and color of red wine anthocyanins, in a wine model solution, were evaluated by spectrophotometric and colorimetric analyses. Phenolic compounds from shavings, mainly ellagitannins, were determined by ultra-high-performance liquid chromatography/mass spectrometry (UHPLC/MS). Antioxidant and copigmentation effects varied depending on the type of shavings (AOW and UOW) and the phenolic concentration (100, 400, and 500 mg/L). Phenolic compounds from shavings improved the color characteristics (darker and more bluish color) and the copigmentation effect of red wine anthocyanins, being UOW a better source of copigments than AOW shavings. The best antioxidant activity was found for the 400 and 500 mg/L model solutions for both types of shavings. Results show a winemaking technological application based on the repurposing of cooperage byproducts, which could improve color and antioxidant characteristics of red wines.

## 1. Introduction

In geographical areas with typical climatological conditions of warm climate, such as Andalucía (Spain), the elevated temperatures make it difficult to obtain high-quality red wines because of color instability over time. In these regions, at the moment of harvesting, different levels of both phenolic and sugar maturity exist. Therefore, copigmentation is affected in the winemaking process because phenolic compound levels are not adequate [1,2]. Phenolic compounds are involved in the copigmentation phenomenon, which is a natural process based on noncovalent complexation between anthocyanins among themselves (self-association), between the central anthocyanin chromophore and aromatic acyl residues covalently linked to their glycosyl moieties (intramolecular copigmentation), or through intermolecular interaction with a wide variety of colorless organic compounds named copigments or copigmentation cofactors (mainly other phenolic compounds). This phenomenon plays a crucial role in the color evolution and stability of young red wines [3,4]. Therefore, an extra contribution of phenolic compounds could solve the color instability problem of red wines.

Several studies have reported that the external addition of phenolic compounds from natural sources, such as byproducts derived from grapes or wood barrels, improves the phenolic content and the color stabilization in wines [5,6,7]. For example, white grape byproducts contain large amounts of phenolic compounds [8] and the addition of seeds from these byproducts during the red wine elaboration increases the phenolic content, the antioxidant activity and the color stabilization [9,10]. In a previous paper, our research group studied the effects on the copigmentation and antioxidant activity of winemaking byproducts (pomace, skins, stems, and seeds). The results indicated that these could improve the color of the anthocyanins and the bioactivity of wines [11]. Moreover, the wine color can be stabilized by the addition of tannins derived from wood [12,13].

Wood shavings are byproducts obtained from the manufacture of wooden barrels that cause environmental and economic problems for cooperages. Therefore, the valorization of those byproducts attracts a great interest [14,15]. Wood shavings are rich in different phenolic acids such as gallic, ellagic, protocatechuic, 4-hydroxybenzoic, vanillic, caffeic, *p*-coumaric, ferulic, syringic and sinapic acids, phenolic aldehydes such as protocatechuic aldehyde, syringaldehyde, coniferaldehyde and sinapaldehyde, and ellagitannins such as vescalagin, castalagin, grandinin, and some roburins [16].

As previously mentioned, phenolic compounds from these byproducts have copigmentation effects and they could be used to solve the problem of color stabilization of red wines. In addition, phenolic compounds are the main contributors to the antioxidant capacity of woods used in cooperage [17]. Therefore, wood shavings could also increase the bioactivity of the wine.

Nowadays, the manufacture of wood barrels generates a high amount of these shavings, which are a source of high added value compounds [14,15,17]. As far as we know, no previous studies have been published regarding wood shavings in order to simultaneously improve antioxidant activity and color stability of red wines. Therefore, it is interesting to carry out studies in this respect.

The aim of this work was, firstly, to evaluate and compare the potential of two different oak wood shavings, American (AOW) and Ukrainian (UOW), as a source of antioxidants and copigments, and, secondly, to ascertain the potential influence of these cooperage byproducts on the antioxidant activity and the color of red wines anthocyanins. For this purpose, the model solutions (in conditions close to those existing in wines) were prepared with phenolic extracts from two type of wood shavings and anthocyanins from red grape skins, and the antioxidant activity, the copigmentation effects and the color of the solutions were measured.

## 2. Materials and Methods

### 2.1. Reagents

Sodium carbonate, sodium acetate, potassium persulphate, 2,4,6-tris(2-pyridyl)-s-triazine (TPTZ) and phosphate-buffered saline (PBS) were purchased from Sigma-Aldrich (Madrid, Spain). Hydrochloric acid, ethanol, glycine, Folin reagent, and iron trichloride (FeCl_3_·6H_2_O) were obtained from Panreac (Barcelona, Spain). ABTS (2,2-azino-bis-(3 ethylbenzothiazolne-6-sulfonic acid) diammonium salt) and Trolox (6-hydroxy-2,5,7,8-tetramethyl-chroman-2-carboxylic acid) were purchased from Fluka (Madrid, Spain).

### 2.2. Samples

Oakwood raw shavings from two oak species classified by their geographical origin were used in this study: American (*Quercus alba*) (AOW) and Ukrainian (*Quercus petraea*) (UOW). Shavings samples, collected as byproducts from the manufacture of non-toasted oak wood barrels, were supplied by the cooperage Salas S.L. (Bollullos Par del Condado, Spain), where these two types of wood are the most abundant.

Usually, barrels used in vinification have different toasting levels, however, these shavings were collected during the process of making barrels with unroasted wood. The shavings were generated by a sawing process of the staves in the longitudinal direction of the fibers. A total of ten samples were collected, grouped, and mixed. Samples were stored in a dry chamber until use.

Additionally, skins of red grapes from Tempranillo variety (*Vitis vinifera* cv. Tempranillo) cultivated in “Condado de Huelva” designation of origin in south-western Spain were used. This grape variety is one of the main red grapes in this region. Grapes were collected at technological ripeness. Skins were manually separated from the pulps and stored at −20 °C until analysis.

### 2.3. Phenolic Extraction

Prior to the extraction process, oak wood raw shavings and red grape skins were freeze-dried in a lyophilizer (Cryodos-80, Telstar Varian DS 102) for 24 h and ground with a mill (IKA A11-B) to obtain a fine and homogeneous powder.

The phenolic extraction from shavings was carried out in a wine-like medium (hydroalcoholic solution: 5 g/L tartaric acid in 12% ethanol, pH = 3.6 and ionic strength adjusted to 0.2 M). Each sample (500 mg of fine powder) was macerated in 10 mL of the hydroalcoholic solution with agitation for 24 h. The mixture was centrifuged at 4500 rpm for 5 min in order to separate the liquid fraction (containing the phenolic compounds extracted) from the solid residue. The solid residue was subjected to the same process twice more (with 1 h of maceration instead of 24 h). The three supernatants (liquid fractions rich in phenolic compounds) were combined and filtered through 0.45 μm filter, thus obtaining the oak phenolic extracts.

The oak phenolic extracts from each type of shavings (AOW and UOW) were analyzed for their phenolic composition (spectrophotometric and chromatographic analysis) and antioxidant activity, and used as a source of copigments and antioxidants for the model solutions.

The extraction of anthocyanins from red grape skins was carried out macerating 5 g of dried and ground skins in 75 mL of the hydroalcoholic solution, following the process previously described for shavings. The anthocyanin solution was analyzed (chromatographic analysis) and used for the model solutions as a source of pigments.

### 2.4. Preparation of Model Solutions

Model solutions were made in a wine-like medium using the skins’ anthocyanin extracts from red grapes and the oak phenolic extracts obtained from wood shavings. For this purpose, each oak phenolic extract (AOW and UOW) was added to the skins anthocyanin extract at three different levels. The final total phenolic content (TPC), being 100, 400, and 500 mg/L, and the final total anthocyanin content (TAC), which was the same in all cases (200 mg/L), were adjusted by dilution with the hydroalcoholic solution. These concentrations were used considering the results of a previous study in model solutions [11].

All model solutions (2 mL) were prepared in triplicate and stored in darkness at 25 °C. After 2 h from their preparation [11], antioxidant activity, color, and copigmentation of model solutions were evaluated. The skins anthocyanin extract, conveniently diluted with the hydroalcoholic solution, was used as the control solution.

### 2.5. Total Phenolic Content

The TPC of oak phenolic extracts from wood shavings was evaluated using the Folin–Ciocalteu assay [18]. Gallic acid was employed as a calibration standard (0–1000 mg/L, y = 0.002x + 0.009, r^2^ = 0.998), and results were expressed as milligrams of gallic acid equivalents per liter of solution (mg GAE/L). All oak phenolic extracts were analyzed in triplicate.

### 2.6. Individual Phenolic Profile

The individual phenolic compounds of the oak phenolic extracts from shavings were determined by ultra-high-performance liquid chromatography (UHPLC), in an Agilent 1290 chromatograph (Agilent Technologies, Palo Alto, CA, USA) equipped with a diode-array detector, following the method previously described [19]. A C18 Eclipse Plus 120 column (1.8 μm, 2.1 × 50 mm) was used. For analysis, samples were filtered through a 0.45 μm pore size membrane filter, and 0.3 μL of sample was injected in the column.

Mass spectrometry (MS) detection was performed in a 3200 Qtrap (ABSCIEX) equipped with an electrospray ionization source and a triple-quadrupole ion trap mass analyzer, which was controlled by Analyst 1.7 software. MS spectra were acquired in the negative ionization modes between *m*/*z* 100 and 2000. The MS detector was programmed to perform a full scan. The experimental conditions of the method were reproduced following a previous study [20]. Phenolic compounds were identified by their retention time, UV-vis spectra, and mass spectra and by comparison with our data library and standards when available. The compounds were quantified from the areas of their chromatographic peaks recorded at 280 nm and results are expressed in percentages.

The individual anthocyanins of the skins anthocyanin extract from red grape were identified by high performance liquid chromatography (HPLC) as previously described [11]. The identity of these compounds was also confirmed by MS. TAC (mg/L) was calculated as the sum of individual anthocyanins contents from a calibration curve of malvidin-3-*O*-glucoside (0–200 mg/L, y = 34.116x + 110.560, r^2^ = 0.999). Individual anthocyanins contents are expressed in percentages.

Three replicates from each sample were analyzed and all the samples and standards were injected three times to obtain the means.

### 2.7. Antioxidant Activity

The antioxidant activity was measured by two spectrophotometric methods: ferric reducing antioxidant power (FRAP) and ABTS, that were performed using the protocols previously described [21,22].

For both assays, different dilutions of each sample were assayed, and the results were obtained by interpolating the absorbance on a calibration curve obtained with the Trolox standard (30–1000 μM). Three independent experiments were performed in triplicate, and the results were expressed as Trolox-equivalent antioxidant capacity (TEAC): μmols Trolox with the same antioxidant capacity as 1 L of solution (μmol TE/L).

### 2.8. Color and Copigmentation Analyses

The whole visible transmission spectrums (380–770 nm, Δλ = 2 nm) of samples were registered with an Agilent UV-vis 8453 spectrophotometer (Agilent Technologies. Palo Alto, CA, USA), using 2 mm path length glass cells. Distilled water was used as a reference blank. The control and model solutions were analyzed by triplicate.

The CIELAB parameters (L*, a*, b*, C*_ab_, and h_ab_) were calculated with software CromaLab^®^ [23], following the recommendations of the Commission Internationale de L’Eclairage [24]: D65 Standard Illuminant, 10° Standard Observer.

Color difference parameter is used to evaluate relationships between visual and numerical analyses. This color difference parameter (ΔE*_ab_) was calculated as the Euclidean distance between two points in three-dimensional space defined by L*, a*, and b*:

ΔE*_ab_ = ((ΔL*)^2^ + (Δa*)^2^ + (Δb*)^2^)^1/2^ (1)

The magnitude of the copigmentation (% copigmentation) was calculated by comparing control solution absorbance (A_0_) and model solution absorbance (A_c_), at 520 nm [25]:

% Copigmentation = [(A_c_ − A_0_)/A_0_] × 100 (2)

Following the methodology previously described [11], diverse color difference formulas were applied in the CIELAB color space.

The total color (E) was assessed as the CIELAB color difference between sample color (L*, a*, b*) and distilled water (L* = 100, a* = 0, b* = 0):

E = ((L* − 100)^2^ + (a* − 0)^2^ + (b* − 0)^2^)^1/2^ (3)

The color variation due to copigmentation was evaluated also by tristimulus colorimetry comparing the total color of the control solution (E_0_) and the total color of the model solutions (E_c_).

[(E_c_ − E_0_)/E_0_ ] × 100 (4)

The color variation induced by copigmentation was assessed as the CIELAB color difference (ΔE*_ab_) between the control solution color (L*_0_, a*_0_, and b*_0_) and the model solutions color (L*_c_, a*_c_, and b*_c_):

ΔE*_ab(c-0)_ = ((L*_c_ − L*_0_)^2^ + (a*_c_ − a*_0_)^2^ + (b*_c_ − b*_0_)^2^)^1/2^ (5)

The relative contribution of lightness (%ΔL*), chroma (%ΔC*_ab_), and hue (%Δh_ab_) to each total color difference induced by copigmentation was calculated as follows:

%ΔL* = ((ΔL*_c-0_)^2^/(ΔE*_ab(c-0)_)^2^) × 100 (6)

%ΔC*_ab_ = ((ΔC*_ab(c-0)_)^2^/(ΔE*_ab(c-0)_)^2^) × 100 (7)

%ΔH = ((ΔH_c-0_)^2^/(ΔE*_ab(c-0)_)^2^) × 100 (8)

ΔH being deduced from ΔE*_ab_, ΔL*, and ΔC*_ab_ values as follows:

ΔH_c-0_ = ((ΔE*_ab(c-0)_)^2^ − ((ΔL*_c-0_)^2^ + (ΔC*_ab(c-0)_)^2^)^1/2^

### 2.9. Statistical Analysis

For the statistical treatment of the data, the Statistica v.8.0 software was used [26]. In order to study significant differences between the different types of model solutions in terms of phenolic composition, antioxidant activity, and color characteristics, a one-way and factorial analysis of variance were carried out using the general linear model procedure (GLM). Tuckey’s test was used to evaluate the significance of the analysis. In addition, correlations between TPC and antioxidant activity values were studied. In all cases, statistically significant level was considered at *p* < 0.05.

Each model solution and each extract were prepared in triplicate (n = 3) and each triplicate was analyzed three times (nine analysis). Results are expressed as mean ± standard deviation (except in percentages, in which values are expressed without deviation).

## 3. Results and Discussion

### 3.1. Phenolic Composition of Oak Phenolic Extracts

The total phenolic contents of oak phenolic extracts from shavings are shown in Table 1.

The oak phenolic extract from UOW and AOW shavings had similar TPC (665.47 ± 80.98 and 556.58 ± 87.94 mg GAE/L, respectively), and significant differences were not found (*p* > 0.05).

In a previous work, the extracts from American woods (*Quercus alba*) had lower total phenolic content than the extracts from two European woods (*Quercus robur* and *Quercus petraea*) [17]. In addition, other authors indicated that American oak woods have a content of total ellagitannins lower than that of European oaks [27]. The major compounds in woods are ellagitannins, which constitute a complex class of polyphenols characterized by one or more hexahydroxydiphenoyl moieties esterified to a sugar [16,17].

The detailed phenolic profiles of oak phenolic extracts from AOW and UOW shavings are shown in Table 1. A total of ten compounds were determined. Regarding phenolic acids, only gallic acid and ellagic acid were detected. Among ellagitannins, four monomers (vescalagin, castalagin, grandinin, and roburin E) and four dimers (roburins A, B, C and D) were identified. These compounds were reported in the literature as important components of oak wood [20,28,29,30].

As is shown in Table 1, the qualitative phenolic profile is the same for the two types of oak phenolic extracts. Vescalagin and castalagin were the most abundant compounds found in both oak phenolic extracts (28.5% and 26.4% for AOW, and 29.2% and 29.7% for UOW, respectively). The rest of ellagitannins showed lower relative proportions, between 3% and 11%. Considering phenolic acids, these compounds were in a lower proportion than all ellagitannins evaluated (<3%). Gallic acid had a higher proportion than ellagic acid in the oak phenolic extracts (2.7% and 3% vs. 0.5% and 0.4%, respectively).

### 3.2. Antioxidant Activity of Oak Phenolic Extracts

Results showed that the antioxidant activity was in accordance with the TPC of each oak phenolic extract, according to the type of wood (Table 2). Antioxidant activity for the oak phenolic extracts from UOW shavings was 7713.61 ± 411.34 and 3809.98 ± 508.80 μmol TE/L, for ABTS and FRAP methods, respectively. These values for oak phenolic extracts from AOW shavings were 6733.79 ± 319.35 and 2385.29 ± 406.12 μmol TE/L, respectively. Significant differences (*p* < 0.05) were found between the antioxidant activity of oak phenolic extracts from UOW and AOW shavings for the FRAP data. However, the antioxidant activity of UOW and AOW determined by the ABTS assay was not significantly different (*p* > 0.05).

The correlation between TPC and antioxidant activity values was evaluated and a high positive and significant correlation (*p* < 0.05) was found: r = 0.87 for the FRAP assay, and r = 0.81 for the ABTS assay. Therefore, the lower FRAP values of the oak phenolic extract from AOW shavings may be due to the TPC of this type of wood. According to other authors, phenols, especially ellagitannins, are mainly responsible for the antioxidant capacity of wood extracts [17,27].

### 3.3. Composition and Antioxidant Activity of Skins Anthocyanin Extract

The total anthocyanin content and the individual anthocyanins of the skins’ anthocyanin extract obtained from Tempranillo grapes are shown in Table 3.

Eleven anthocyanins were identified and quantified, constituting a TAC of 253.79 ± 15.96 mg/L. Malvidin-3-*O*-glucoside was the predominant anthocyanin (44.7%), followed by malvidin-3-*O*-acetyl-glucoside (16.4%), which is in agreement with those reported in Tempranillo and Syrah skins [11,31].

Values of antioxidant activity were 6919.83 ± 106.29 and 4950.64 ± 61.56 μmol TE/L, for ABTS and FRAP assays, respectively (Table 2). Although other phenolic compounds from grape skins could have antioxidant activity, it has been indicated that the anthocyanin fraction from Tempranillo skins was the greatest contributor to antioxidant activity [32].

### 3.4. Antioxidant Activity of Model Solutions

Results of antioxidant activity of the control solution and the model solutions with different TPC are shown in Table 4.

As can be observed, differences in the antioxidant activity of the model solutions were found depending on the type of wood (from UOW and AOW) and the phenolic concentration level (100, 400, and 500 mg/L). Antioxidant activity was higher for all concentration levels than for control (Table 4).

Regarding ABTS assay, the same pattern was observed for model solutions from UOW and AOW shavings. Control model solution (TAC = 200 mg/L) had the lowest antioxidant activity (1300.89 ± 37.33 μmol TE/L). The antioxidant activity for the 100 mg/L model solutions was not significantly different (*p* > 0.05) from that of the control solution (1396.81 ± 33.74 and 1489.61 ± 224.16 for UOW and AOW, respectively). An increase in the antioxidant activity for the 400 and 500 mg/L model solutions was observed (2037.66 ± 33.74 and 1839.07 ± 150.59 for UOW, respectively; 2248.57 ± 0.97 and 1707.33 ± 274.98 for AOW, respectively), showing significant differences with the control solution and the 100 mg/L model solution (*p* < 0.05).

Antioxidant activity measured by FRAP method also was higher for the 400 and 500 mg/L model solutions than for the 100 mg/L model solution and the control solution (Table 4) (*p* < 0.05). A correlation analysis between TPC and antioxidant activity confirmed that the greater antioxidant effects in the 400 and 500 mg/L model solutions than in 100 mg/L and control solutions are due to their higher TPC.

Significant differences were not found between model solutions from AOW and UOW shavings for the antioxidant activity.

### 3.5. Color and Copigmentation Effect in Model Solutions

**Figure 1:**
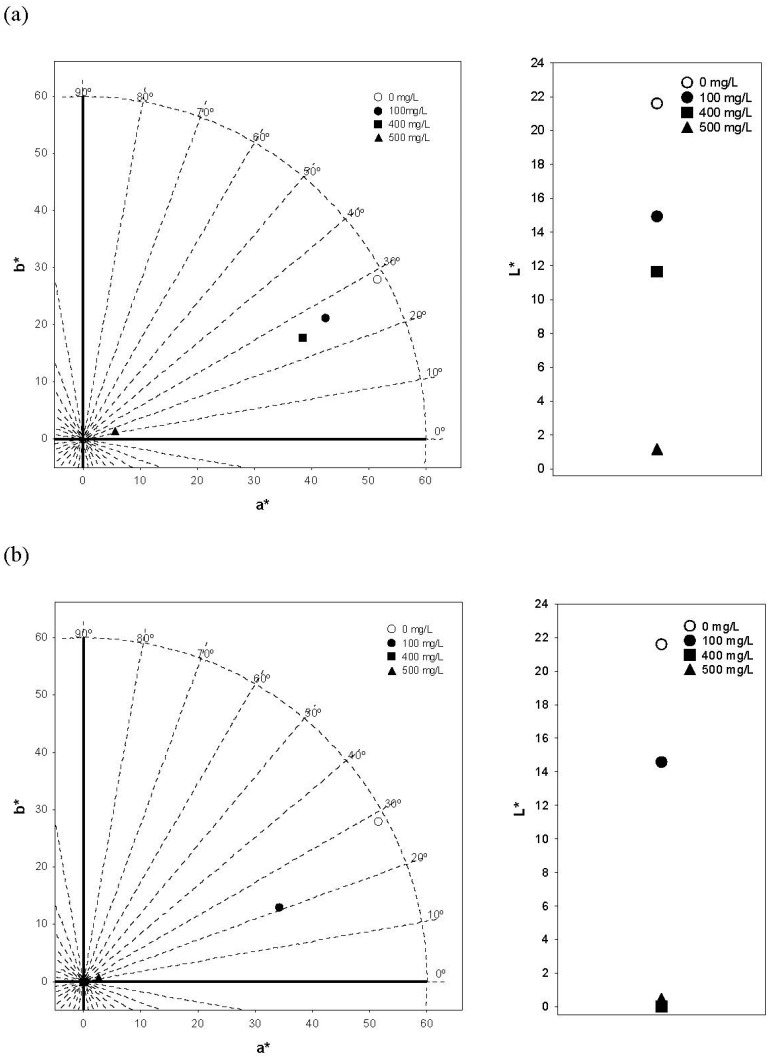
(a*b*) Color diagram and lightness (L*) of the control solution and the model solutions with different TPC from (**a**) American (AOW) and (**b**) Ukrainian (UOW) shavings.

A color analysis (CIELAB space) of the model solutions and control solutions was performed. Figure 1 depicts the location of the samples within the (a*b*) diagram and lightness values (L*).

The CIELAB color parameters of the solutions were influenced by the type of wood (AOW and UOW) and the TPC of the model solutions. Lightness, chroma and hue values (L*, C*_ab,_ and h_ab_, respectively) of the model solutions decreased with the increase of the TPC. In general, solutions in the presence of the copigments exhibited darker and more bluish color than the control solution. These results show that an important part of the expression of the color of the anthocyanins could be due to the copigmentation phenomenon in which they are involved, leading to changes at both quantitative and qualitative levels in the color of the wine.

The effect of the type of wood (AOW and UOW shavings) and TPC on color characteristics of the model solutions was evaluated. Significant differences between the two types of wood were found (*p* < 0.01), as well as between model solutions with different TPC (*p* < 0.001). The results indicated a strong influence (*p* < 0.001) of TPC on all colorimetric parameters (L*, C*_ab_, h_ab_). However, the type of wood influenced (*p* < 0.05) all colorimetric variables except L*.

In the model solutions from AOW, the colorimetric parameter values (L*, C*_ab_, h_ab_) were lower for all concentration levels (100 mg/L: 14.9, 47.4 CIELAB units, 26.5°; 400 mg/L: 11.7, 42.5 CIELAB units, 23.6°; 500 mg/L: 1.2, 5.7 CIELAB units, 14.7°, respectively) than for the control solution (21.6, 58.5 CIELAB units, and 28.5°, respectively) (Figure 1a). These differences were significant (*p* < 0.05) between the control solution and the 500 mg/L model solution for L*, and between the 500 mg/L model solution and all the other model solutions for C*_ab_.

Differences were also found between the control solution and UOW model solutions. UOW model solutions showed lower chroma (C*_ab_), lightness (L*) and hue (h_ab_) values than the control solution (Figure 1b). The decrease in C*_ab_ and h_ab_ was more noticeable for the 400 and 500 mg/L model solutions (400 mg/L: 0.1 CIELAB units and 14.2°; 500 mg/L: 2.6 CIELAB units and 16.0°, respectively), and presented significant differences with the control solution (*p* < 0.05).

**Figure 2:**
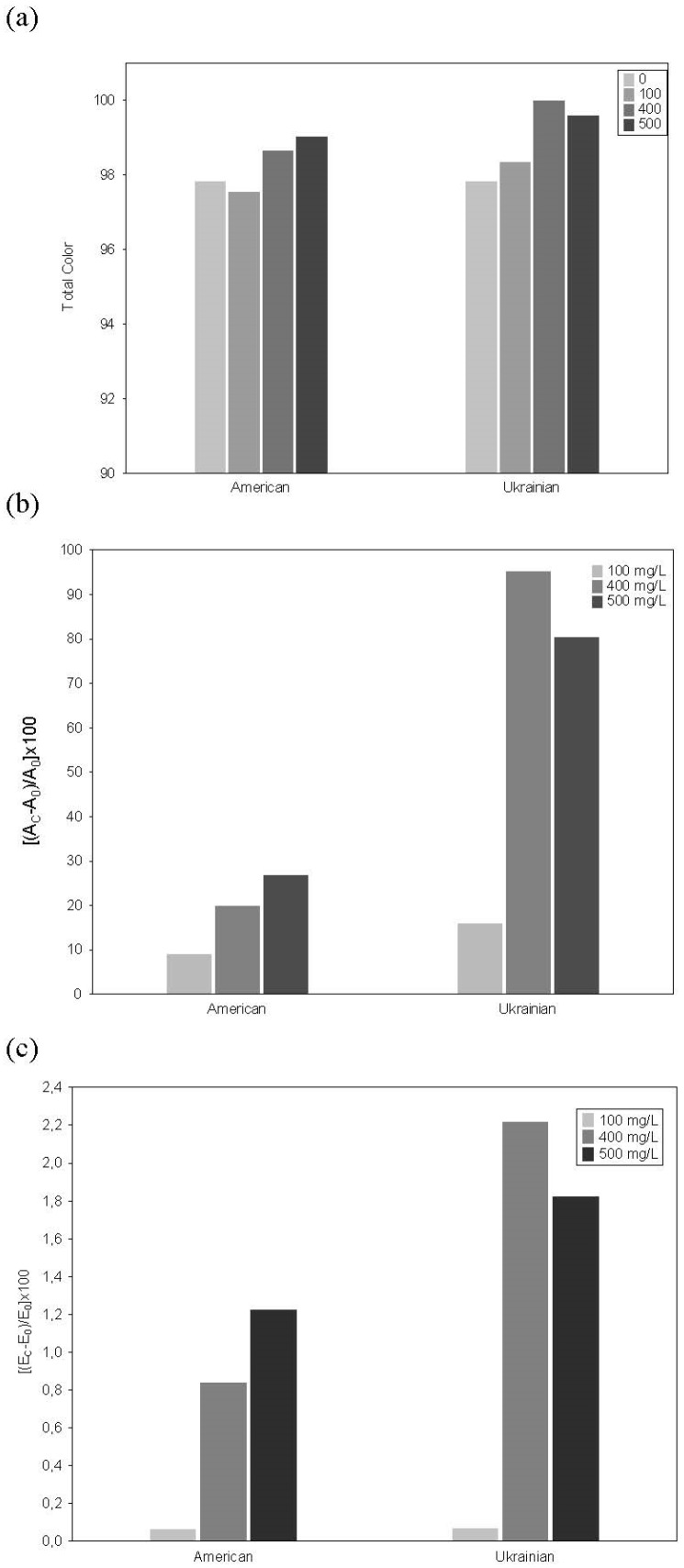
(**a**) Total color, (**b**) magnitude of copigmentation evaluated by the absorbance at 520 nm (**c**) and by tristimulus colorimetry of the control solution and model solutions with different TPC.

The total color and the effect of copigmentation of the model solutions with increasing concentrations of phenolic compounds are shown in Figure 2.

Intermolecular copigmentation was observed in the model solutions: The control solution showed a hyperchromic shift of its λ_max_ (520 nm) and an increase of its initial total color (97.8 CIELAB units) (Figure 2a).

The copigmentation magnitude evaluated by the absorbance at 520 nm and by tristimulus colorimetry was different depending on the type of wood and the TPC (Figure 2b,c). It was observed that the copigmentation effects of model solutions from UOW (15.93%, 95.20%, and 80.32%, for 100, 400 and 500 mg/L, respectively) were higher than of solutions from AOW (8.90%, 19.90%, and 26.82%, for 100, 400 and 500 mg/L, respectively) (Figure 2b). The effect was significantly different (*p* < 0.05) for the 400 mg/L model solution.

**Figure 3:**
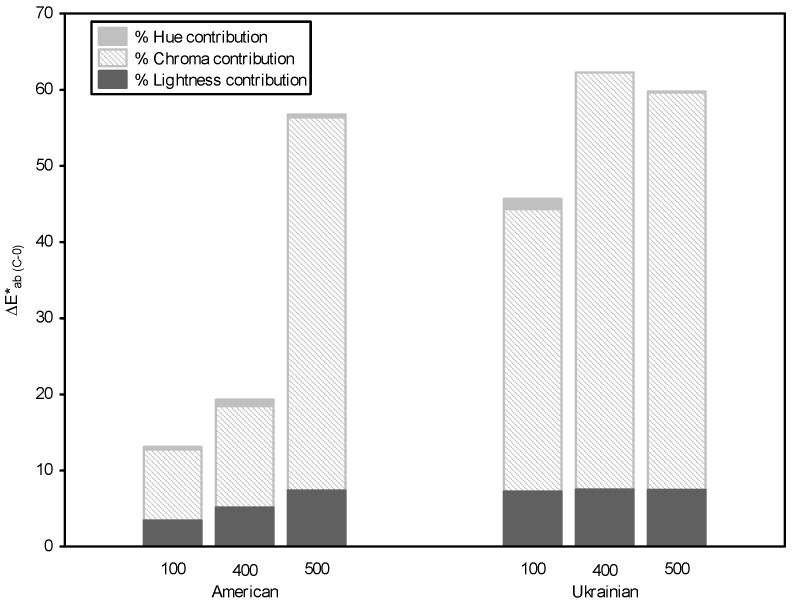
Total color difference induced by copigmentation (∆E*_ab (C-0)_), between the color of the control solution and the color of the model solutions with different TPC from American (AOW) and Ukrainian (UOW) shavings. Relative contribution of chroma, lightness and hue (%∆*C*, %∆*L*, %∆*h*) to the color difference.

The color changes that provide color information related to visual perception were carried out by means of color differences calculation between the control solution and model solutions with different TPC (Figure 3). Moreover, the relative contribution of each colorimetric parameter (%∆*L*, %∆*C* and %∆*h*) to color difference permits an objective comparison of the colorimetric effects among samples. Results showed that the color changes of the model solutions from AOW and UOW shavings at all the concentrations were visually perceptible (∆E*_ab_ > 3, [33]). It can be observed that, in general, the relative contribution of lightness and chroma to color differences was greater than that of hue (%∆*L* = 23% and 16%; %∆*C* = 75% and 74%; %∆*h* = 2% and 9%, for AOW and UOW model solutions, respectively).

Our results indicate that phenolic compounds extracted from oak byproducts improve the color characteristics and copigmentation effect of red anthocyanins in model solutions. These results are in accordance with the ones reported by some authors about the influence of some compounds from wood, like ellagic acid and ellagitannins, on the copigmentation and color of wine [34,35,36,37,38]. UOW shavings are a better source of colorless copigments than AOW shavings. The difference between both kinds of wood (AOW and UOW) could be due to American oak wood is characterized by a lower total ellagitannin content than the European oak [39].

## 4. Conclusions

Oak phenolic extracts obtained from oak wood shavings were a great source of phenolic compounds, mainly ellagitannins, which had antioxidant and copigmentation effects. Extracts from the two types of oak wood improved the antioxidant activity and the color of red wine anthocyanins in the model solutions. These effects depended on the oak phenolic extract (AOW and UOW) and the phenolic concentration level in the model solution (100, 400, and 500 mg/L).

In general, the best antioxidant activity and color characteristics were found for the 400 and 500 mg/L model solutions for AOW and UOW. Regarding the type of wood, UOW and AOW model solutions had similar antioxidant activity values. However, the model solutions from UOW had a darker and more bluish color, and a higher magnitude of copigmentation than AOW.

The results indicate that cooperage byproducts are a good natural source of antioxidants and copigments, providing Ukrainian shavings better effects on anthocyanins color stability. Therefore, the use of these byproducts in red wine vinification could improve color and bioactivity of wines. These results may be of interest to the wine industry and the cooperages implied in the manufacture of wooden barrels.

## Figures and Tables

**Table 1:** Total phenolic content and distribution of ellagitannins and phenolic acids identified by ultra-high-performance liquid chromatography/mass spectrometry (UHPLC/MS) in the oak phenolic extracts from American (AOW) and Ukrainian (UOW) shavings.

			**AOW**	**UOW**
**TPC** (mg/L)			556.58 ± 87.94 ^a^	665.47 ± 80.98 ^a^
**Compound**	**MW**	**MS**	**Relative Proportion** (%)	
**Ellagitanins**				
*Monomers*				
Vescalagin	934	933, 466	28.5	29.2
Castalagin	934	933, 466	26.4	27.9
Grandinin	1066	1065	4.8	4.7
Roburin E	1066	1065	5.7	6.1
*Dimers*				
Roburin A	1851	924, 616	11.0	9.9
Roburin D	1851	924, 616	7.4	7.2
Roburin B	1983	990, 660	9.3	8.7
Roburin C	1983	990, 660	3.8	3.4
**Phenolic acids**				
Ellagic acid	302	301	0.5	0.4
Gallic acid	170	169	2.7	3.0

Each value represents mean (n = 3) ± standard deviation. For total phenolic content (TPC), values followed by the same letter in the same row indicate no significant differences (*p* > 0.05). MW: molecular weight, MS: Mass spectrometric.

**Table 2:** Antioxidant activity of the oak phenolic extracts from American (AOW) and Ukrainian (UOW) shavings and of the skins anthocyanin extract from red grapes.

	AOW	UOW	Skins Anthocyanin Extract
**ABTS** (µmol TE/L)	6733.79 ± 319.35 ^a^	7713.61 ± 411.34 ^a^	6919.83 ± 106.29
**FRAP** (µmol TE/L)	2385.29 ± 406.12 ^a^	3809.98 ± 508.80 ^b^	4950.64 ± 61.56

Each value represents mean (n = 3) ± standard deviation. Values followed by different letters in the same row indicate significant differences (*p* < 0.05).

**Table 3:** Total anthocyanin content and distribution of anthocyanins identified by HPLC/MS in the skins’ anthocyanin extract from red grapes.

Anthocyanin	Relative Proportion (%)
Delphinidin-3-*O*-glucoside	6.1
Cyanidin-3-*O*-glucoside	1.2
Petunidin-3-*O*-glucoside	9.2
Peonidin-3-*O*-glucoside	7.4
Malvidin-3-*O*-glucoside	44.7
Petunidin-3-*O*-acetyl-glucoside	1.6
Peonidin-3-*O*-acetyl-glucoside	1.1
Malvidin-3-*O*-acetyl-glucoside	16.4
Petunidin-3-*O*-*p*-coumaroyl-glucoside	1.6
Peonidin-3-*O*-*p*-coumaroyl-glucoside	2.5
Malvidin-3-*O*-*p*-coumaroyl-glucoside	8.1
TAC (mg/L)	253.79 ± 15.96

**Table 4:** Antioxidant activity of the control and model solutions with different TPC from American (AOW) and Ukrainian (UOW) shavings.

	AOW		UOW	
	ABTS	FRAP	ABTS	FRAP
**Control solution**	1300.89 ± 37.33 ^a^	509.45 ± 41.32 ^a^	1300.89 ± 37.33 ^a^	509.45 ± 41.32 ^a^
**Model solution**				
100 mg/L	1489.61 ± 224.16 ^a^	608.05 ± 2.85 ^a^	1396.81 ± 33.74 ^a^	716.63 ± 76.88 ^a^
400 mg/L	2248.57 ± 0.97 ^b^	927.43 ± 40.97 ^b^	2037.66 ± 33.74 ^b^	847.93 ± 87.92 ^b^
500 mg/L	1707.33 ± 274.98 ^b^	851.27 ± 153.28 ^b^	1839.07 ± 150.59 ^b^	1006.12 ± 43.28 ^b^

Each value represents mean (n = 3) ± standard deviation. Values followed by different letters within a column indicate significant differences (*p* < 0.05). Results are expressed as µmol TE/L.
